# Heart Rate Variability as Indicator of Clinical State in Depression

**DOI:** 10.3389/fpsyt.2018.00735

**Published:** 2019-01-17

**Authors:** Ralf Hartmann, Frank M. Schmidt, Christian Sander, Ulrich Hegerl

**Affiliations:** Department of Psychiatry and Psychotherapy, University Hospital Leipzig, Leipzig, Germany

**Keywords:** heart rate variability, depression, biomarker, diagnostics, antidepressants, response parameter, trait marker, state marker

## Abstract

**Background:** Depression is a severe disease with great burdens for the affected individuals and public health care systems. Autonomic nervous system (ANS) dysfunction indexed by measures of heart rate variability (HRV) has repeatedly been associated with depression. However, HRV parameters are subject to a wide range of multi-factorial influences and underlying mechanisms in depression are still unclear. HRV parameters have been proposed to be promising candidates for diagnostic or predictive bio-markers for depression but necessary longitudinal design studies investigating the relationship between HRV and depression are scarce.

**Methods:** The sample in this study consisted of 62 depressive individuals without antidepressant medication prior to assessment and 65 healthy controls. Fifteen minute blocks of resting ECGs were recorded 1–2 days before onset of antidepressant treatment and 2 weeks thereafter. The ECGs were pre-processed to extract inter-beat-intervals. Linear and non-linear methods were used to extract HRV parameters. ANOVAS were performed to investigate group differences between depressive patients and healthy controls. Associations between the change in severity of depression and HRV parameters were assessed in a repeated measurements design.

**Results:** Analyses revealed HRV parameter differences between the groups of depressive patients and healthy controls at baseline. Further results show differences in HRV parameters within subjects after 2 weeks of antidepressant treatment. Change in HRV parameter values correlated with changes in symptom severity of depression.

**Discussion:** The current results provide further insight into the relationship between HRV parameters and depression. This may help to underpin utilization of HRV parameters are bio-maker for disease state in depression. Results are discussed within a theoretical framework to link arousal and ANS regulation in depression.

## Introduction

Depression is a severe disease with high prevalence (1–3) and an elevated risk for recurrence and chronicity (4). It is a major source of burden of disease and contributes significantly to years lived with disability (5). Repeated findings of increased occurrence of cardio-vascular disease among patients with Major Depressive Disorder [MDD, (6–11)] have drawn attention to autonomous regulation of the heart rate as a potential pathophysiological mechanism in depression (12–19). Consequently, altered Autonomous Nervous System (ANS) regulation of the heart rate indicated by measures of heart rate variability (HRV) has repeatedly been observed in depression across the life span (15, 20–22). Heart rate is typically assessed by electrocardiogram (ECG) and different procedures have been established to describe HRV and derive parameters from different time-length ECG signals (23–27). A compelling body of research indicates a reduced HRV indexed by lower values of SDNN, RMSSD and HF power and increased values of LF power for patients with depression in comparison to healthy controls (15, 28–32). LF/HF Ratio has repeatedly been found to be decreased in depression (15) but recent research leaves doubts about interpretation of the LF/HF ratio (33–35). Some HRV parameters have been used to separate patients with MDD and healthy controls (31, 36). Moreover, reduced HRV has been associated with severity of depression and parameters derived from HRV have been applied to delineate the severity of depression or even changes in symptom severity (13, 28, 37). Inconsistent results exist concerning the association between HRV and the response to antidepressant treatment (38–41). Hitherto, research on longitudinal monitoring of HRV and symptom severity of depression is scarce. Though some studies found changes in HRV to normalize along with improvement in severity of depression during antidepressant treatment (15, 38, 42) findings for specific HRV parameters remain inconsistent. Studies investigating changes in HRV parameters and severity of depression over the course of time are of importance for a better understanding of the relationship between HRV and disease state when HRV is supposed to be used as a diagnostic or predictive bio-marker for depression. Bio-markers for depression might be helpful in clinical treatment and disease management (43). Previous attempts to utilize HRV parameters as bio-markers for depression are promising (44–46). To underpin these attempts we aim to examine in a repeated measurements design the relationship between HRV and symptom severity of depression.

This study investigates the relationship between HRV parameters and the severity of depression in a repeated measurements design. First aim was to identify HRV parameters which could serve as diagnostic markers to separate patients with MDD from healthy controls at baseline. A further aim of this study was to examine if patients with MDD show normalization of HRV parameters parallel to an improvement in symptom severity of depression and if changes in HRV correlate with changes in symptom severity of depression over the course 2 weeks of antidepressant treatment. This may augment understanding if HRV parameters may serve as indicators for the presence of MDD or change in symptom severity of depression over the course of time.

## Materials and Methods

### Sample

All data presented in this work were gathered alongside a study on brain arousal regulation as response predictor for antidepressant therapy in Major Depressive Disorder (MDD) using Electroencephalography (EEG) signals (47). ECG was obtained in parallel to the EEG recording at two time points: prior to antidepressant treatment (T1) and 14 ± 1 days following onset of antidepressant treatment (T2). The total sample of the aforementioned study (47) comprised 65 unmedicated patients with a diagnosis of MDD and 65 healthy controls. The patient subsample consisted of depressed in- and outpatients consecutively recruited between 2012/02 and 2015/01 from the Department of Psychiatry and Psychotherapy of the University Hospital Leipzig. Individuals had to meet the inclusion criteria: age ≥ 18 years; a diagnosis of MDD with a current depressive episode with a base-line score ≥ 10 in the HDRS-17. Exclusion criteria were defined to meet the EEG study requirements: use of a centrally active medication (including anti-depressants) within 14 days prior to the T1 assessment. Other exclusion criteria were acute risk of suicidal tendencies; somatic mental disorders; illegal drugs and/or alcohol abuse within the past 6 months; schizophrenia, schizotypal and delusional disorders; a history of head injury with loss of consciousness exceeding 1 h; seizure disorder; acute or chronic infection and major somatic disorders. The diagnosis of a unipolar depressive disorder was given by a clinical professional and was substantiated by the Structured Clinical Interview for DSM-IV Axis I (SCID-I) prior to the T1 assessment. The subsample of healthy controls consisted of 65 sex- and age-matched non-depressed controls selected from a database consisting of EEG-recordings from community volunteers recruited via announcements in the local newspapers, University's intranet and internet. For detailed information upon sample characteristics see Schmidt et al. (47).

For the present analyses, three individuals of the original patient subsample had to be excluded because of missing ECG recordings at T1. Therefore, the total sample of the present study comprised 62 unmedicated patients with a diagnosis of MDD (depressive sample, DEP) and 65 healthy controls (HC). The HC sample was only assessed at T1 therefore ECG signals of healthy controls were not available at T2 for direct comparisons between groups. To evaluate normalization of HRV parameters of the DEP sample we used the T1 HRV parameters of the HC sample. Within the DEP sample, 8 individuals had to be excluded from T1/T2 comparisons due to missing ECG recordings at T2.

The study was performed according to the Helsinki Declaration and approved by Leipzig University Ethics Committee (#278-11-22082011).

### Procedures

Assessments of symptoms and severity of MDD and measurements of ECG signals took place before the onset of antidepressant treatment (T1) and 2 weeks following the onset of medication (T2). Standard antidepressant treatment was carried out according to an in-house treatment algorithm at the Department of Psychiatry and Psychotherapy of the University Hospital Leipzig which includes antidepressant medication as first line treatment. At both assessments symptom severity of depression was assessed with the 17 Item version of the Hamilton Depression Rating Scale [HDRS-17, (48)], and Inventory of Depressive Symptomatology [IDS-C, (49)] by a trained rater using a structured interview guideline (50). The Beck Depression Inventory II [BDI-II, (51)] was issued for self-rating of symptoms and severity of MDD. All ECG measurements were carried out as additional measurements alongside EEG recordings. EEG and ECG signals were measured in the same room between 8:00 a.m. and 2:00 p.m. Within patients, time of recording was not allowed to vary more than± 1 h between T1 and T2. For each time point individuals were instructed to relax with eyes closed in a semi-recumbent position and not to fight a possibly occurring urge to fall asleep. ECG signals were recorded with a 40 channel QuickAmp amplifier (Brain Products GmbH, Gilching, Germany) with 2 leads placed on the left forearm at a sampling rate of 1 kHz.

### HRV Analyses

BrainVision Analyzer 2 (Brain Products GmbH, Gilching, Germany) was used to analyse all ECGs for interbeat intervals (IBIs). After automated R-wave peak detection of the QRS complex (for details see 26) all data sets were manually scanned for ectopic heartbeats and artifacts were removed. After this procedure RR distances in milli seconds were calculated. Artiifact (52) and Kubios (53) were used to obtain HRV parameters from the resulting tachograms. Both applications were used to double check results for accuracy and consistency. Time domain HRV parameters were calculated by linear methods, frequency domain HRV parameters were calculated by using Spectral Analyses (e.g., Fast Fourier Transformation) and non-linear HRV parameters were calculated by using Poincare plots. All HRV parameters were selected based on literature (23, 25, 27) and are presented in Table 1.

**Table 1:** HRV parameters derived from time-domain, frequency-domain and non-linear procedures, abbreviations and descriptions.

**Parameter**	**Description**	**Unit**	**ANS branch**
**TIME DOMAIN**			
SDNN	Standard deviation of NN intervals	ms	SNS
RMSSD	Root mean square of successive RR interval differences	ms	PNS
pNN50	Percentage of successive RR intervals that differ by more than 50 ms	%	SNS, PNS
**FREQUENCY DOMAIN**			
LF power	Relative power of the low-frequency band (0.04–0.15 Hz)	ms^2^, %, n.u.	SNS
HF power	Relative power of the high-frequency band (0.15–0.4 Hz)	ms^2^, %, n.u.	PNS
LF/HF Ratio	Ratio of LF-to-HF power	%	SNS, PNS
**NON-LINEAR**			
SD1	Poincaré plot standard deviation vertical the line of identity	ms	Short term flexibility of ANS
SD2	Poincaré plot standard deviation along the line of identity	ms	Long term flexibility of ANS
SD1/SD2 Ratio	Ratio of SD1 and SD2	ms	

*ANS, Autonomous Nervous System; SNS, Sympathetic Nervous System; PNS, Parasympathetic Nervous System; Hz, Hertz; ms, milli seconds; ms^2^, milli seconds squared; n.u., normalized units; NN Intervals, interval between two normal R-peaks; RR intervals, interval between two R-peaks; for further information see Task Force of the European Society of Cardiology, and the North American Society of Pacing and Electrophysiology (23)*.

### Statistical Analyses

ANOVAs were performed to investigate group differences at baseline. Simple regressions and binary logistic regressions were performed to predict severity of depression or group assignment (DEP vs. HC) according to data level. To investigate differences between time points repeated measures ANOVAs were performed with HRV parameters as within subject factors and age, sex and heart rate as between subject factors. Effect sizes were calculated using Eta square. For approximation of normalization of HRV parameter the T1 values of HC were carried forward to T2. Change in symptom severity between time points was defined as reduction in sum score of HDRS-17, BDI-II and IDS-C of 30 and 50% at T2 compared to T1. To estimate change in HRV parameters we calculated absolute value differences between T1 and T2. Principle component analyses were performed to assess subtypes of depression (atypical subtype, melancholic subtype) based on respective single item ratings of BDI-II and HDRS-17 separately [see (54)]. Sample was stratified for severity of depression (a) based on cut-offs for BDI-II with values from 14 to 19 for mild depression, values from 20 to 28 for moderate depression and values above 29 for severe depression (51) and (b) HDRS-17 [9–16 = mild depression, 17–24 = moderate depression, ≥ 25 = severe depression (55)]. All Statistical analyses were performed using SPSS (SPSS 24, IBM, Chicago, IL, United States) and the significance level was set to a *p* < 0.05.

## Results

### General

Total sample mean age was 36.99 (*SD* = 12.108) ranging from 19 to 61 years. The total sample comprised of 62 males and 65 females. We found no violation of normal distribution for age within groups. Furthermore, we found no significant differences between groups for age [*t*_(125)_ = −0.066, *p* = 0.948] and sex (*X*^2^ = 0.378, *df* = 1, *p* = 0.538). We collapsed data across sex and age for subsequent analyses. There were no differences between groups for heart rate [*F*_(1, 125)_ = 1.087, *p* = 0.299] and Mean RR interval [*F*_(1, 125)_ = 0.770, *p* = 0.382].

### Group Differences in HRV at Baseline

To investigate which HRV parameters separate between depressive patients (DEP) and healthy controls (HC) we conducted an ANOVA for mean differences between both groups at T1. Group differences in HF power in % [*F*_(1, 125)_ = 15.029, *p* = 0.000, Eta^2^ = 0.115] were highly significant. Group differences in SD1 [*F*_(1, 125)_ = 4.909, *p* = 0.029, Eta^2^ = 0.034], RMSSD [*F*_(1, 125)_ = 4.909, *p* = 0.029, Eta^2^ = 0.038] and LF power in % [*F*_(1, 125)_ = 4.605, *p* = 0.23, Eta^2^ = 0.04] were significant. Age and sex as confounding variables were not significant. Means and Standard Errors for time domain, frequency domain and non-linear parameters for DEP vs. HC at T1 and for repeated measurements (DEP_T1_ vs. DEP_T2_) are presented in Table 2. Furthermore, we performed binary logistic regressions analyses to predict group assignment to HC and DEP from HRV parameters. HF power in % predicted group assignment to the HC vs. DEP sample (*X*^2^ = 15.517, *df* = 1, *p* = 0.000) explaining 62.2% of all variance. Also LF power in % (*X*^2^ = 5.137, *df* = 1, *p* = 0.023, explained variance = 51.2%) and SD1 (*X*^2^ = 6.154, *df* = 1, *p* = 0.013, explained variance = 51.2%) significantly predicted group assignment. The simple regression analyses to predict symptom severity from HRV parameters within the depressive sample did not show significant results.

**Table 2:** Heart Rate Variability in healthy controls (HC) and MDD patients (DEP) and MDD patients before (T1) and after 2 weeks of antidepressant treatment (T2).

	**HC**	**DEP**		**DEP**		
	**T1/N = 65**	**T1/N = 62**		**T1/N = 53**	**T2/N = 53**	
	**Mean (SE)**	**Mean (SE)**	***p***	**Mean (SE)**	**Mean (SE)**	***p***
**TIME DOMAIN**						
SDNN in ms	50.464 (3.831)	48.030 (6.059)	0.732	41.489 (2.168)	40.525 (2.611)	0.666
RMSSD in ms	41.547 (5.323)	28.872 (1.778)	**0.029**	28.859 (1.952)	32.318 (3.413)	0.264
pNN50 in %	16.133 (2.390)	10.301 (1.688)	0.050	10.202 (1.865)	13.206 (2.617)	0.203
**FREQUENCY DOMAIN**						
LF in Hz	0.075 (0.003)	0.074 (0.003)	0.761	0.074 (0.003)	0.078 (0.003)	0.394
LF power in %	51.283 (2.055)	43.511 (2.716)	**0.023**	44.385 (2.910)	46.076 (2.533)	0.513
LF power in n.u.	56.173 (2.330)	59.932 (2.205)	0.244	59.038 (2.443)	49.943 (2.889)	**0.001**
HF in Hz	0.250 (0.007)	0.244 (0.007)	0.519	0.241 (0.008)	0.250 (0.008)	0.291
HF power in %	40.782 (2.305)	28.593 (1.939)	**0.000**	29.695 (2.115)	47.396 (2.932)	**0.000**
HF power in n.u.	43.721 (2.323)	39.989 (2.197)	0.246	40.877 (2.434)	50.001 (2.885)	**0.001**
LF/HF Ratio	2.093 (0.350)	2.071 (0.197)	0.955	2.008 (0.209)	1.561 (0.243)	0.052
**NON-LINEAR**						
SD1 in ms	29.394 (3.766)	20.426 (1.258)	**0.029**	20.417 (1.381)	22.864 (2.415)	0.264
SD2 in ms	46.274 (3.793)	57.509 (10.038)	0.289	47.399 (3.380)	38.293 (3.003)	**0.001**
SD1/SD2 Ratio	1.935 (0.092)	3.135 (0.679)	0.075	2.466 (0.137)	1.901 (0.090)	**0.000**

*Hz, Hertz; ms, milli seconds; ms^2^, milli seconds squared; n.u., normalized units; SDNN, Standard deviation of NN intervals; RMSSD, Root mean square of successive RR interval differences; pNN50% = Percentage of successive RR intervals that differ by more than 50 ms; LF power = Relative power of the low-frequency band (0.04–0.15 Hz); HF power = Relative power of the high-frequency band (0.15–0.4 Hz); SD1 = Poincaré plot standard deviation vertical the line of identity; SD2 = Poincaré plot standard deviation along the line of identity. Significant p-values are highlighted in bold*.

### Changes in HRV and Depression Severity

Assessing changes in symptom severity of depression between T1 and T2 within the depressive sample (DEP), the Repeated Measurements ANOVAs revealed highly significant differences with moderate to high effect sizes in self-rated (BDI-II) as well as observer-rated (HDRS, IDS-C) instruments. Results are displayed in Table 3. Repeated Measurements ANOVAs revealed changes in HRV parameters within the depressive sample (DEP, for results see Table 2). Differences between time points for HF power in % [*F*_(1, 52)_ = 33,180, *p* = 0.000, Eta^2^ = 0.39] and SD1/SD2 Ratio [*F*_(1, 52)_ = 14,773, *p* = 0.001, Eta^2^ = 0.221] were highly significant with moderate effect size. Differences between time points for HF power in n.u. [*F*_(1, 52)_ = 12,375, *p* = 0.001, Eta^2^ = 0.192] and SD2 [*F*_(1, 52)_ = 13,019, *p* = 0.001, Eta^2^ = 0.2] were significant with moderate effect size. In addition differences between time points for LF power in n.u. [*F*_(1, 52)_ = 14,773, *p* = 0.001, Eta^2^ = 0.19] were significant with moderate effect size. Age and heart rate were not identified as confounders. We found a significant interaction of sex and HF power in % [*F*_(1, 51)_ = 5.075, *p* = 0.029, Eta^2^ = 0.091]. The difference in HF power values between sexes at T1 [*M*_*males*_ = 26.975(*SE* = 2.885), *M*_*females*_ = 32.742 (*SE* = 3.053)] was more pronounced at T2 [*M*_*males*_ = 38.376 (*SE* = 3.634), *M*_*females*_ = 57.498 (*SE* = 3.846)]. To appraise normalization of HRV parameters we conducted ANOVA for group differences between T1 HC and T2 DEP. No significant group differences were found. Furthermore, we aimed to test our hypothesis that change in HRV parameters correlate with change in symptom severity of depression after 2 weeks of antidepressant treatment. Point-Biserial correlations were run to determine the relationship between changes in HRV parameters and changes in symptom severity of depression indexed by reduction in sum scores of HDRS, BDI-II and IDS-C. Only results for Delta HF power were significant, age and sex as confounding variables were not significant (for results see Table 4).

**Table 3:** Change in Symptom severity of MDD patients before (T1) and after 2 weeks of antidepressant treatment (T2) as indexed by HDRS, BDI-II and IDS-C.

	**T1 (before medication)**		**T2 (after medication)**			
	**Mean**	**SE**	**Mean**	**SE**	***p***	**Eta^**2**^**
BDI-II	29.579	1.514	24.140	1.855	**0.000**	0.289
HDRS	21.934	0.798	15.541	0.961	**0.000**	0.471
IDS-C	36.317	1.408	27.450	1.689	**0.000**	0.346

*BDI-II, Beck Depression Inventory II; HDRS, Hamilton Depression Scale; IDS-C, Inventory of Depressive Symptomatology Clinician Version. Significant p-values are highlighted in bold*.

**Table 4:** Change in HF power (Delta HF power in %) and change symptom severity of MDD before and after antidepressant treatment, correlations *r*_*pb*_ and *p*-values, *N* = 53.

**Variables**	**Delta HF power**	***p***
50% ^a^ HDRS	−0.337	**0.021**
50% ^a^ BDI-II	−0.133	0.388
50% ^a^ IDS-C	−0.218	0.146
30% ^*b*^HDRS	−0.273	0.063
30% ^*b*^BDI-II	−0.115	0.458
30% ^*b*^IDS-C	−0.301	**0.042**

a *Reduction in values between T1 (before medication) and T2 (after 14 days of medication) ≤ 50%*.

b *Reduction in values between T1 and T2 ≤ 30%. Significant p-values are highlighted in bold*.

Furthermore, we conducted exploratory analyses to investigate relationships between HRV parameters and other variables, such as severity of depression and subtypes of depression (atypical vs. melancholic subtype), which did not reveal significant results.

## Discussion

Heart rate variability has been in the focus of research for bio-markers of depression for a long time (46). Promising attempts to utilize HRV parameters as diagnostic or predictive bio-markers of depression have been presented. However, there is still a lack of studies investigating the relationship between HRV and severity of depression in longitudinal designs which is important if HRV is supposed to be used as bio-marker for depression. The purpose of this study was to provide further insight into the relationship of HRV and severity of depression in a repeated-measurements design.

### Group Differences in HRV at Baseline

Aim of this study was to identify HRV parameters that may serve as diagnostic maker to separate between depressive patients and healthy controls prior to antidepressant treatment. Our results show significant differences between groups of patients with depression and healthy controls. The depressive sample displays lower HF power values compared to healthy controls. This result can be interpreted as a reduced ability of the para-sympathetic nervous system to regulate the heart rate via vagal activity [e.g., (56, 57)]. SD1 results show significantly reduced values in the depressive patients sample compared to healthy controls. This result may be interpreted as diminished short-term flexibility of the ANS to adapt to changing environments and tasks [i.e., (58–60)]. These findings present further evidence to previous findings of reduced HRV in depressive patients (15, 31). HF power was able to predict group assignment (MDD patients and healthy controls) with acceptable accuracy but regression analyses revealed no statistically significant relationship between HRV and symptom severity. Depressive individuals may be limited in their ability to adapt their ANS activity to challenging environments and fail to evoke adaptive resources (58, 61). This may result in phenotypical depressive symptoms like exhaustion, fatigue, stress-reactivity and disrupted sleep patterns (62). We also found significant results for RMSSD, LF power and SD1. Those parameters may serve as diagnostic bio-marker for pathophysiologic differences between MDD and healthy controls. But since underlying mechanisms that motor those parameters remain unclear interpretation should be treated with caution.

### Changes in HRV and Depression Severity

Another aim of this study was to investigate if patients with MDD show normalization of HRV parameters parallel to an improvement in symptom severity of depression after 2 weeks of antidepressant treatment. Differences between time points for parameters reflecting severity of depression (HDRS-17, BDI-II and IDS-C) were significant. Individuals had lower levels of symptom severity after 2 weeks of antidepressant treatment assessed with both self-rated and observer-rated instrument even though the period between time points was very short and we did not expect individuals to remit fully from MDD symptom severity. We interpreted results as a reduction (i.e., improvement) in symptom severity of depression over the course of 2 weeks of antidepressant treatment, even though individuals partly remained diseased. We expected HRV parameters to normalize over the course of 2 weeks of antidepressant treatment. Within the group of depressive patients the differences between time points for HF power, LF power, SD2 and SD1/SD2 Ratio were significant. Values of HF power and Non-linear parameters increased over time and were even more pronounced in women than man and may be interpreted as an increase in vagal activity [see (25)]. Although LF power n.u., SD2 and SD1/SD2 Ratio differences between time points were significant, we refused to interpret these parameters due to heterogeneous findings at baseline. Furthermore, HRV parameter values at T2 did not differ from HRV parameter values of healthy controls at T1. This may be interpreted as a normalization of HRV parameter values in correspondence with improvement in symptom severity. To further investigate the relationship between HRV parameters and symptom severity we aimed to test the hypothesis that changes in HRV parameters correlate with changes in symptom severity of depression. We found reciprocal correlations for observer-rated instruments HDRS-17 (at 50% reduction) and IDS-C (at 30% reduction) for HF power but with a small effect size. We interpret these findings so that increase of HF power values correlates with reduction in symptom severity.

Our results support our hypothesis of normalization of HRV parameters and improvement of symptom severity of depression in correspondence to an increase in HRV parameters values. This may be due to a link between both, nonetheless the exact mechanism behind the link remains unclear. A possible mechanism could be a regulation deficit between the two branches of the Autonomous Nervous System. A similar dysregulation has been proposed as explanation for findings of hyperstable vigilance found in MDD (63), as well as changes in the arousal regulation during therapy to relate to changes in depression severities (47). However, explained variance is still small and we suggest further research to unveil relationship between HRV and depression and the influence of confounding variables if HRV parameters shall be utilized as diagnostic or predictive bi-markers for depression.

## Limitations

Our study faces some limitations which may impeach our interpretations. First of all our data shown here is based on ECG signals that were obtained parallel to EEG recordings and therefore the ECG signals were not suited to extract reliable time-domain measurements (e.g., RMSSD) and we did not control for in/expiration (23). Since overall data acquisition was laid out with accurate and ecologically valid and sample size was good we decided to investigate the ECG data set. Furthermore, our samples were not randomized and we did not have ECG signals for controls at T2. Since we only had a 2 weeks period between measurements long term effects may not be delineated. Our sample size of depressive patients at T1 was probably too small to reveal significant correlations of HRV parameters and measurements indexing symptom severity of depression. At last, though results for change in HRV parameters values and change in symptom severity were significant the amount of explained variance was small. Therefore, interpretation und clinical relevance may be limited.

## Conclusion

The aim of this study was investigate the relationship between HRV parameters and symptom severity of depression in a repeated measurements design to provide further insight into this relationship which is of importance if HRV parameters shall be utilized as diagnostic or predictive bio-markers of depression. We interpret our results as confirmation of our hypothesis. Patients with MDD show decreased HRV compared to healthy controls before antidepressant treatment. Furthermore, patients with MDD show normalization of HRV parameters parallel to an improvement in symptom severity of depression over the course 2 weeks of antidepressant treatment and change in HRV parameter values correlates with change in symptom severity of depression after 2 weeks of antidepressant treatment. Though HF power seems to be a promising indicator for disease state in depression further research is needed to investigate the relationship between HRV parameters and symptom severity of depression and confounding variables in longitudinal study designs.

## Data Availability Statement

Original data is available upon request.

## Ethics Statement

The study was performed according to the Helsinki Declaration and approved by Leipzig University Ethics Committee (#278-11-22082011). Ethical Committee at the Medical Faculty, Leipzig University, Käthe-Kollwitz-Straße 82, 04109 Leipzig.

## Author Contributions

FMS and UH were responsible for the planning and conduct of the original study (47). FMS recruited the study participants, supervised the EEG/ECG recordings and was responsible for the collection of clinical data. CS and FMS were responsible for data management and the pre-processing of the EEG files. RH performed the HRV analyses as well as the statistical analyses and was the main author of the manuscript. All authors participated substantially in writing the manuscript.

### Conflict of Interest Statement

The authors have the following conflicts to declare: Within the last 3 years, UH was an advisory board member for Lundbeck, Takeda Pharmaceuticals, Servier and Otsuka Pharma; a consultant for Bayer Pharma; and a speaker for Bristol-Myers Squibb, Medice Arzneimittel, Novartis and Roche Pharma. The remaining authors declare that the research was conducted in the absence of any commercial or financial relationships that could be construed as a potential conflict of interest.
